# Biological invasions: a field synopsis, systematic review, and database of the literature

**DOI:** 10.1002/ece3.431

**Published:** 2013-01-10

**Authors:** Edward Lowry, Emily J Rollinson, Adam J Laybourn, Tracy E Scott, Matthew E Aiello-Lammens, Sarah M Gray, James Mickley, Jessica Gurevitch

**Affiliations:** 1 Department of Ecology & Evolution, Stony Brook UniversityStony Brook, New York, 11794-5245; 2 Department of Botany, The Academy of Natural Sciences of Drexel UniversityPhiladelphia, Pennsylvania, 19103; 3 Department of Biology, Unit of Ecology & Evolution, University of Fribourgch. du Musée 10, CH-1700, Fribourg, Switzerland; 4 Department of Ecology and Evolutionary Biology, University of ConnecticutStorrs, Connecticut, 06269-3043

**Keywords:** Biological invasions, Charles Elton, disturbance, EICA, enemy escape, invasion hypothesis, systematic review

## Abstract

Species introductions of anthropogenic origins are a major aspect of rapid ecological change globally. Research on biological invasions has generated a large literature on many different aspects of this phenomenon. Here, we describe and categorize some aspects of this literature, to better understand what has been studied and what we know, mapping well-studied areas and important gaps. To do so, we employ the techniques of systematic reviewing widely adopted in other scientific disciplines, to further the use of approaches in reviewing the literature that are as scientific, repeatable, and transparent as those employed in a primary study. We identified 2398 relevant studies in a field synopsis of the biological invasions literature. A majority of these studies (58%) were concerned with hypotheses for causes of biological invasions, while studies on impacts of invasions were the next most common (32% of the publications). We examined 1537 papers in greater detail in a systematic review. Superior competitive abilities of invaders, environmental disturbance, and invaded community species richness were the most common hypotheses examined. Most studies examined only a single hypothesis. Almost half of the papers were field observational studies. Studies of terrestrial invasions dominate the literature, with most of these concerning plant invasions. The focus of the literature overall is uneven, with important gaps in areas of theoretical and practical importance.

## Introduction

**Figure 1:**
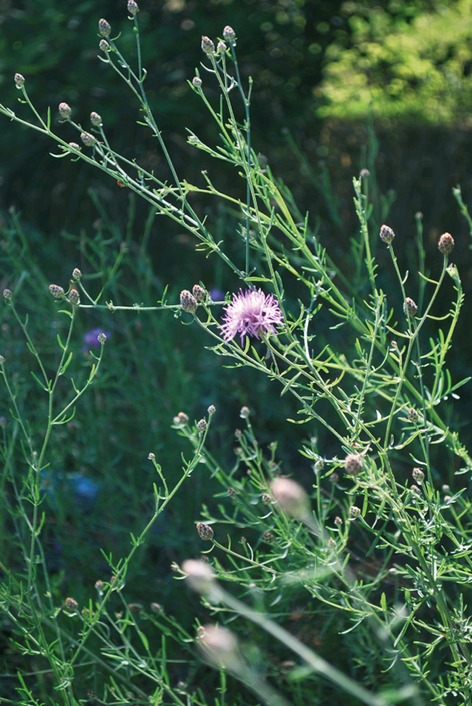
(photo #941) *Centaurea stoebe* L. spp. *micranthos* (Gugler), formerly known as *C. maculosa*, is an invasive plant that has dominated large areas of rangeland in the intermountain western U.S. after being introduced to North America in the late 19th century from Europe, where it is native. It has recently gone from being naturalized to becoming highly invasive in the northern Great Lakes region of the midwestern U.S., and has shown signs of becoming invasive in the eastern U.S., where it has also been naturalized since the late 19th century. Photo by J. Gurevitch taken in eastern Long Island, N.Y.

The literature on biological invasions is enormous; it has grown rapidly since the mid-twentieth century as scientists, managers, policy makers, and the public have become increasingly aware of the many applied issues of managing invasive species, as well as the fundamental ecological questions raised by biological invasions. This body of scientific information on biological invasions addresses many different questions, and varies greatly in scope and focus. A goal of many of these papers has been to attempt to explain biological invasions by posing hypotheses regarding the invasive species, the invaded communities, and their interactions, and there have been a large number of experimental studies that have tested these hypotheses. Others are about the impacts of invasion, control of invasives, or other topics. (Our research group, for instance, is studying Centaurea stoebe L. ssp. micranthos, a European native plant invasive and spreading in various regions of North America; Fig. 1).

Our primary goal was to evaluate what has been studied regarding the causal factors by which species invade novel environments, and the ecological impacts of biological invasions. In order to assess the current state of knowledge, we carried out a field synopsis and a systematic review of this literature. The purpose of the field synopsis was to map and categorize the scope of available information (and what is not known) from the literature addressing a fundamental understanding of biological invasions. The systematic review addressed the state of our knowledge about the mechanisms that permit species to invade novel environments. We carried this out by attempting to identify and characterize the literature, including what hypotheses have been tested, and what organisms and systems have been studied. A secondary goal of our work was to create a publicly accessible database of this literature for future research. We did not attempt to quantify or analyze the outcomes and conclusions of these papers here; rather, our goal is to gain a better understanding of what has been studied. Future research – our own and that of others – will be needed to address and quantify the outcomes of the research covered in this literature database.

The purpose of categorizing studies was to map the literature. In other words, we address a very basic, almost elementary question: what has been published on this topic? What we know depends on what has been studied. If no scientific information exists on a question (in published or unpublished form), we cannot answer the question scientifically. Mapping where we have good information and where we have gaps is essential for making progress. We point out that categorizing studies does not constitute a vote count. A vote count depends on the statistical significance of the outcomes of significance tests. In a vote count, one amasses a body of literature on a question (e.g., do invasive plants have negative effects on natives?) and then counts up the number of “ayes” and “nays” based on the significance tests in each paper, then presumably conclude that if the ayes outweigh the nays, the effect is real, and if there are many more ayes than nays, that it is an important effect. There are well-known statistical reasons why vote-counts are not a reliable approach and can produce uninformative, misleading, and biased results (e.g., Gurevitch and Hedges 1999). Although some other reviews in this field have used vote-counting (e.g., Hayes and Barry 2008; Pyšek et al. 2012), we did not do that here. We are not considering study outcomes, and do not make any comparisons based on counting up numbers of statistically significant outcomes.

Field synopses and systematic reviews are two current approaches for research synthesis taken largely from other disciplines and not yet used widely in ecology. A field synopsis is a literature review in which all relevant information is systematically and objectively gathered on a broad topic (e.g., Khoury et al. 2009). Field synopses have only recently been introduced in the biomedical literature, and have to date been applied or initiated to broadly identify human genomic and genetic associations with disease (e.g., schizophrenia, Allen et al. 2008; melanoma, Chatzinasiou et al. 2011). While the parameters for carrying out field synopses are still being defined, at a minimum, a field synopsis must follow rigid methodological guidelines designed to make the literature review complete, unbiased, objective, transparent, and repeatable. They are often too large and broad in scope to be combined with formal meta-analysis (i.e., quantitative synthesis).

Systematic reviews are, in contrast, very widely used and formalized in the biomedical literature and have become the standard way that reviews are carried out. About 2500 formal systematic reviews are published in the medical literature each year, and that number is accelerating (Moher et al. 2006). They are also very widely used in the social sciences. A systematic review uses clearly and explicitly stated search criteria (see Appendix 1) to comprehensively identify the research bearing on a specific question (Littell et al. 2008). In many fields, the elements required for publication of a systematic review are specified very explicitly, and publication of predefined protocols for conducting the systematic review prior to its inception are either required or encouraged (Cook et al. 1997; Liberati et al. 2004, PLOS Medicine Editors 2011). The Cochrane Library for systematic reviews and protocols in medicine states,

*A systematic review attempts to identify, appraise and synthesize all the empirical evidence that meets prespecified eligibility criteria to answer a given research question. Researchers conducting systematic reviews use explicit methods aimed at minimizing bias, in order to produce more reliable findings that can be used to inform decision making (Cochrane Library, 2011)*.

The protocol should include a well-defined question, state the search criteria, outline the procedure for conducting a thorough search (ideally using several databases), and have clearly stated and specific inclusion/exclusion criteria.

Systematic reviews are often, but not necessarily, combined with quantitative synthesis – meta-analysis – of the research results, although either can be carried out without the other. A major goal of systematic reviewing is to bring scientific methodology to the review process. Ideally, by using clearly defined protocols, the review process can be both repeated by others and more efficiently and accurately updated in the future (Littell et al. 2009). Systematic reviews have been introduced to the ecological literature relatively recently (e.g., Stewart et al. 2005, 2007; Pullin and Stewart 2006; Kettenring and Reinhardt Adams 2011), largely in the conservation and applied ecology literature, and the terms and modern formal methodology for systematic reviews remain unfamiliar to many ecologists. As in the other fields in which they have become adopted, systematic reviews offer numerous advantages in accuracy and reduction of bias over narrative reviews and expert assessments of the literature.

With the exception of some very recent formal systematic reviews (e.g., Ferrer et al. 2011; Parr and Gibb 2007), meta-analyses in ecology (other than conservation and applied ecology) have ranged from those that come close to systematic review methodology (albeit without formally including all of its elements) to those that either do not identify how papers were searched or appear arbitrary in their selection criteria (e.g., “data from all papers known to the authors on Topic X were included.”). These approaches are not systematic or replicable and may be subject to bias and incompleteness.

The field synopsis we report here included studies that investigated biological invasions in natural systems. The systematic review concerned a subset of these studies, focusing on the literature explaining why some species are invasive and some communities are invasible, as well as that addressing fundamental questions in ecology and evolution using the phenomenon of biological invasions (e.g., what determines species range limits or the number of species that can coexist in a community).

Since the seminal book by Elton (1958), narrative reviews and meta-analyses on the mechanisms of biological invasions have shaped our thinking about biological invasions in sometimes profound ways, as is the case for the highly cited papers by Parker et al. (1999) and Sakai et al. (2001), and more recent reviews by Sax et al. (2007), Fridley et al. (2007), and Pyšek et al. (2006, 2008). It was our goal to gain a more comprehensive overview of the literature on biological invasions, as well as to update these older reviews. The scope of many invasion reviews has, however, often been limited in various ways; e.g., Pyšek et al. (2012) emphasized highly cited papers, examining studies cited 30 or more times, while Cadotte et al. (2006) reviewed studies that analyzed at least 100 species. Many narrative reviews are limited to a particular invasive taxon, either more broadly to plants (Richardson et al. 2000; Mitchell et al. 2006) or animals (Snyder and Evans 2006), or to a single functional group or species (e.g., zebra mussels, Karatayev et al.^1997^; ants, Wetterer et al. 2006) or they focus on specific geographic regions (e.g., Foxcroft et al. 2010), habitats (e.g., Van Auken 2000; Weis 2011), or on particular aspects of invasions such as ecological or economic impacts (e.g., Mack and D'Antonio 1998; Kenis et al. 2009). Other reviews have emphasized more specific questions, for example, focusing on efforts to quantify the prediction of the success of invasive plants and birds (e.g., Kolar and Lodge 2001), impacts of invasive plants (Vilà et al. 2011; Pyšek et al. 2012), or comparisons of native and invasive species in more limited subsets of species and restricted regions (e.g., Blackburn and Duncan 2001; Cadotte and Lovett-Doust 2001; Allen et al. 2006). So, in addition to relying upon formal systematic review methodology, our research synthesis is more comprehensive than previous reviews.

## Methods

We began with the same literature search for both the field synopsis and systematic review. The systematic review was a subset of the literature gathered in the field synopsis, which was examined in greater detail. We initiated a literature search for both the field synopsis and systematic review using the ISI Web of Science database and search engine by employing the following search string to identify relevant papers by topic (i.e., using key words):

Topic = (invasi* OR invader OR alien OR exotic OR ruderal OR weed OR non-native OR introduced OR naturaliz) AND topic = (plant OR invertebrate OR ecolog* OR evolut* OR marine OR terrestrial OR freshwater OR aquatic) NOT Topic = (cancer* OR cardio* OR surg* OR carcin* OR engineer* OR operation OR medic* OR crop OR rotation OR ovar* OR polynom* OR purif* OR respirat* OR “invasive technique”).

Next, we limited our database to relevant fields of study by using the “refine” function in the Web of Science to exclude non-relevant subjects such as medicine, agriculture, engineering, astronomy, or physics. We only searched for English language publications. We did not attempt to redefine “invasive” or “invaded”, but left those categorizations and definitions to the authors of the published papers (i.e., we accepted authors' categorization of species as invasive). The initial search included records from 1911 to June 6, 2010. The search was updated on September 29, 2011.

Ideally, field synopses and systematic reviews should search several databases. Because of the large scope of our review, we were not able to do so. As an alternative, we used the search engine SCOPUS to analyze a more limited sample of papers, to determine the extent to which the results would vary with the search engine and to gauge the inclusiveness of our search results from the Web of Science database. The search and exclusion options are not identical between the two databases, but we used the same search terms. We carried out the SCOPUS search on March 8, 2011. Unlike Web of Science, SCOPUS does not include a categorical exclusion feature (i.e., ability to exclude categories such as cancer studies). The records resulting from the SCOPUS search were compared with our Web of Science records by comparing the primary author, first characters of the title, source title, volume, issue, and beginning page number of the record (using a program we wrote), and identical records were discarded. We then narrowed the results to a subset of studies, those on field experiments, to compare with the Web of Science results by including only those articles identified in the SCOPUS search that had “field” or “experiment” in the abstract. For these papers, we categorized the focus of the work, the invasion hypotheses examined, invasive species identities, trophic levels, locations (to country and state), ecosystems, and biomes.

### Field synopsis

We next used the title and abstract (when available) of each paper identified above to assess if the study was relevant according to the criteria below. Further selection was carried out by examining the text of the articles. We defined relevant studies as those concerned with fundamental understanding of biological invasions and we excluded research on agricultural systems, studies concerned primarily with chemical or biological control or management, methods for the eradication of invasive species, papers recording the identification and location of invaders, those focused on predicting potentially invasive species, invasive pathogens, and on the economic impacts of invasions.

We then categorized the studies by date and research focus. The research foci were papers concerned with invasion hypotheses, fundamental questions in ecology and evolution, studies on impacts of invasions, and combinations of one or more of these categories. For subsets of the papers first identified, we had two readers make eligibility and categorization decisions; these were checked, discussed, and rectified until readers were trained. All decisions were reviewed by EL.

### Systematic review

The systematic review was a more detailed analysis of a subset of the papers identified in the field synopsis. We excluded papers concerned with invasion impacts. Studies were then categorized as follows: by type of research, invasive species being studied, trophic level of the invader, invaded ecosystem and biome, and hypothesis being evaluated (detailed in Appendix 2). For studies carried out in the field or in gardens, we identified the location of the study where possible (i.e., where the invasion was located), by country (and state if relevant) and latitude/longitude (when reported). Recent papers reviewing invasive species research (e.g., Inderjit et al. 2005; Catford et al. 2009) have enumerated the common hypotheses attempting to explain biological invasions, and for those papers whose focus was on testing invasion hypotheses, we relied on the lists of hypotheses in these reviews to categorize the hypotheses being tested in the literature (Appendix 3).

### Database creation

We developed a database using R (software by R Development Core Team 2011) and RMySQL (James and DebRoy 2012), importing initial results from Web of Science or SCOPUS. We developed a web-based interface for entering data we collected from each source. The data are available in Appendices 4-6.

## Results

### Field synopsis

#### Number of studies and dates published

**Figure 2:**
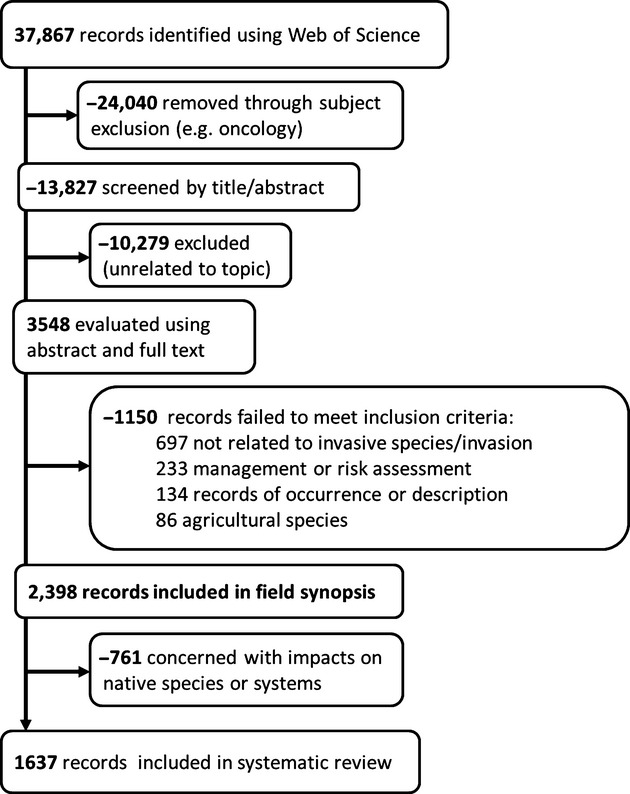
Flow chart detailing the process of record collection and study elimination for the field synopsis and systematic review.

We initially identified 37,563 studies using our search terms; just over 24,000 of these were removed using the “refine” function in Web of Science to exclude papers from other disciplines (Fig. 2). Almost 14,000 studies were then evaluated following our selection criteria using titles and abstracts; over 10,000 of these did not meet our selection criteria and were excluded (e.g., they were not about biological invasions, but concerned structural engineering issues, or were reports of the occurrence of cancer metastasis, fundamental mathematical problems or chemical methods of weed control). Of the remaining 3548 studies, 1150 were excluded after evaluation of the full text of the paper; 233 of these were concerned with management or risk assessment, 134 were strictly descriptive and/or records of occurrence only, 86 were agricultural, and 697 were not related to the topic or were excluded for other reasons (e.g., record #180, which is a publication describing how to design a college course about invasive species, or record #218, which includes the topic “biological invasions” in the abstract, but only addresses general questions of ecosystem function in a system without invaders; this type of erroneous inclusion was not uncommon).

The first study identified in the initial search was published in 1916, and the first relevant study included in the field synopsis was published in 1966. The field synopsis thus identified 2398 relevant studies published between 1966 and September 29, 2011. These included studies concerning invasion hypotheses, fundamental questions, and impacts of invasions.

**Figure 3:**
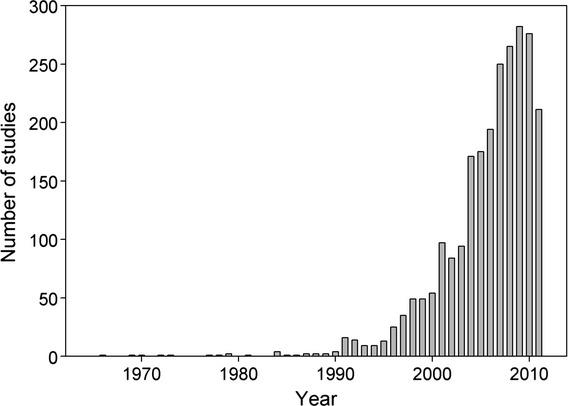
The number of studies published per year included in the field synopsis. The most recent year (2011) only included records included in the database through September (journals published at different dates in September will vary in their inclusion in the database) and indexed on the Web of Science as of September 2011.

Only small numbers of papers matched our search criteria prior to 1990, with 0–2 studies per year from 1966 to 1990 except for four in 1984 (Fig. 3). Four papers were published in 1992, and in 1991, that number quadrupled to 16 papers, and by 1997, it more than doubled to 35 papers. The first review papers were published in 1992 (four papers). Publications continued to accelerate, reaching 171 in 2004, 250 in 2007, and almost 300 papers per year in 2009 and 2010 (the latest years for which we have complete counts). The acceleration in papers published may be slowing, but many papers continue to be published (Fig. 3).

#### Study focus and type of research

**Figure 4:**
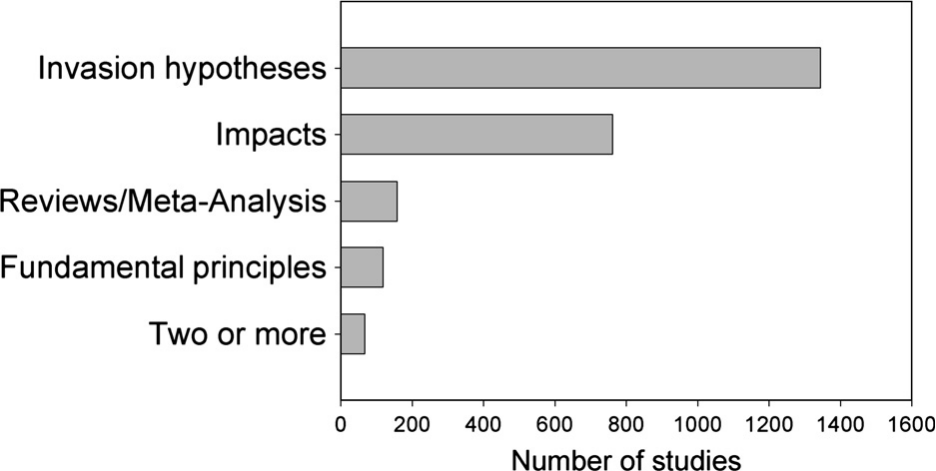
The focus of the publications in the field synopsis. We defined three possible foci: (1) Investigating a hypothesis concerning a biological invasion, (2) evaluating the impacts of a biological invasion, or (3) using a biological invasion as a model system for investigating fundamental ecological questions. A publication may include more than one focus.

A large majority (1405) of the studies we categorized were concerned with explaining causes of biological invasions, while a smaller, but still substantial number of studies (761) were primarily concerned with documenting or testing the impacts of invaders (Fig. 4). Those studies that seek to explain causes of invasions do so by implicitly or explicitly testing or examining hypotheses for the success of the invaders, typically in particular systems. They may explicitly state that they are testing a particular named hypothesis (e.g., EICA) or they may implicitly evaluate an explanation for invasion without formally stating it as a hypothesis for invasion (e.g., a particular trait may be held responsible for the success of an invasion, or a characteristic of the invaded environment may be the explanation for an invasion). We did not distinguish between implicit and explicit tests of invasion hypotheses. Far fewer studies on biological invasions focused on fundamental ecological or evolutionary questions. The remaining papers were reviews, or addressed hypotheses as well as impacts and/or fundamental questions.

### Systematic review

#### Categorization of study type

**Figure 5:**
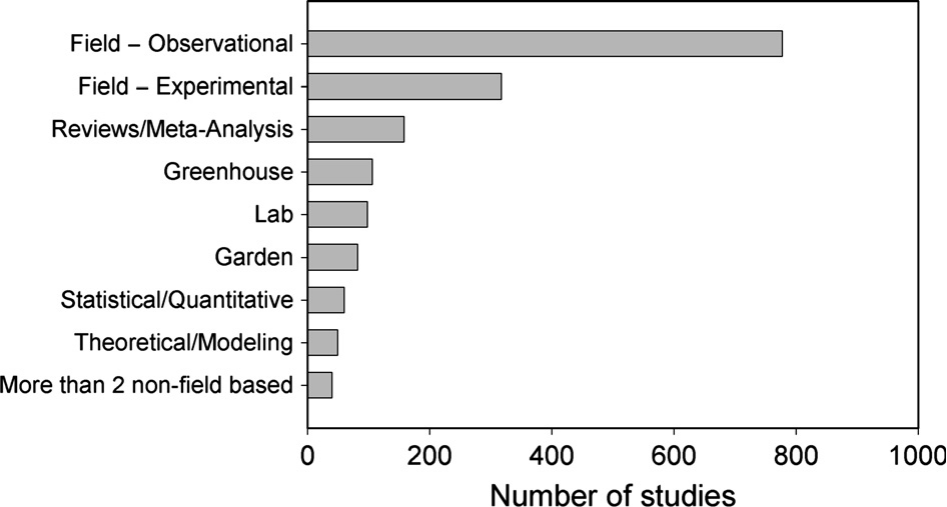
The type of research methods employed in the studies in the systematic review. A description of the categories can be found in the Methods and in Appendix 2. This and all remaining figures refer to the systematic review outcomes.

Field observational studies were by far the most common type of research, representing 46% of the studies (Fig. 5). Field experimental studies, the next most common study type, comprised 18% of the published papers. Fewer experimental studies were carried out in controlled or semi-controlled environments (lab, greenhouse, or garden), and fewer still involved only statistical analyses or theoretical research. Of the 1637 papers categorized, 84% were observational or experimental empirical studies, while 16% were reviews, meta-analyses, statistical analyses, or theoretical/modeling studies. We did not further categorize the reviews, but carried out an examination of the characteristics of the empirical studies to provide an overview of this literature.

#### Geographic location of research

**Figure 6:**
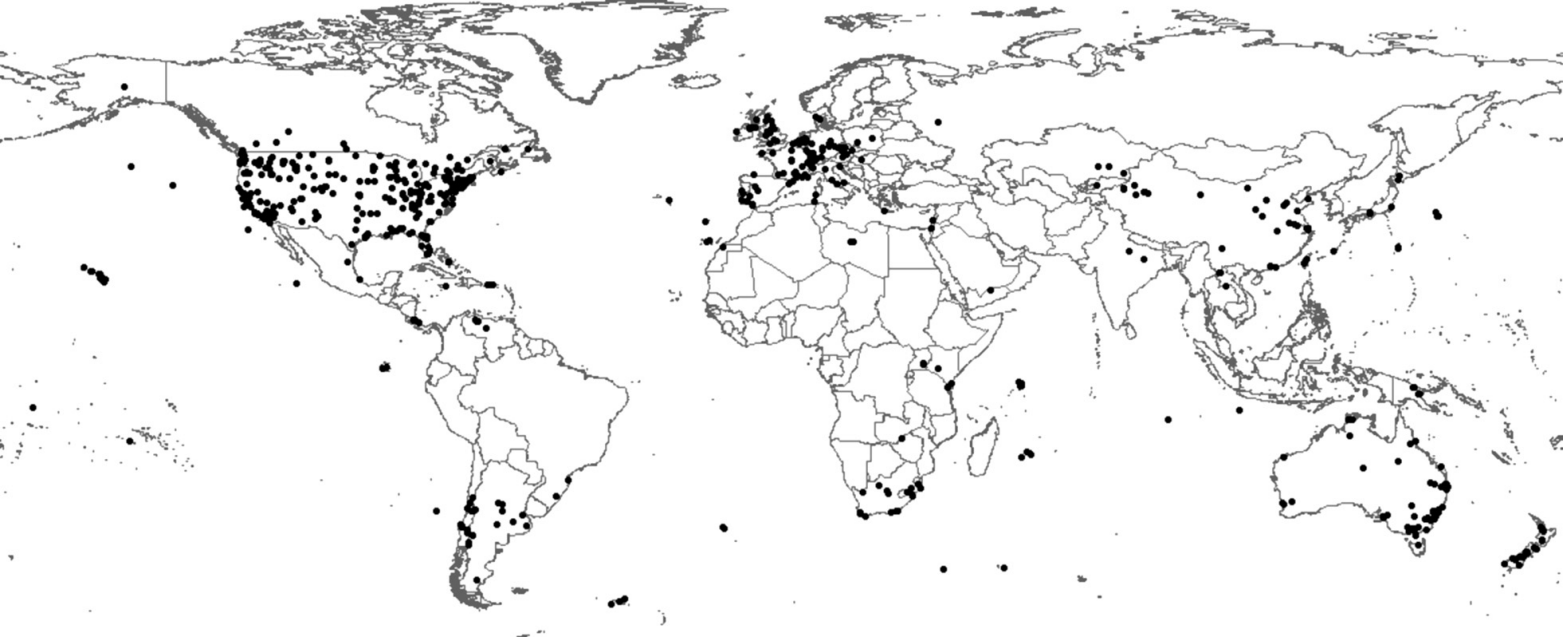
The locations of the studies included in the systematic review that were explicitly specified in the publications. These included 831 locations in 704 publications (more than one location per publication could potentially be included). Latitudes and longitudes were not recorded for studies published from 06/10 to 09/11 (our updated results).

We were able to identify the locations of 703 experimental and observational studies carried out in the field (Fig. 6). Although this research was very widely distributed globally, studies were clustered in North America, Western Europe, eastern Australia, New Zealand, and Hawaii, with smaller clusters in South Africa, temperate South America, and China, and scattered studies elsewhere. We found a dramatic dearth of studies in the tropics.

#### Trophic levels and systems studied

**Figure 7:**
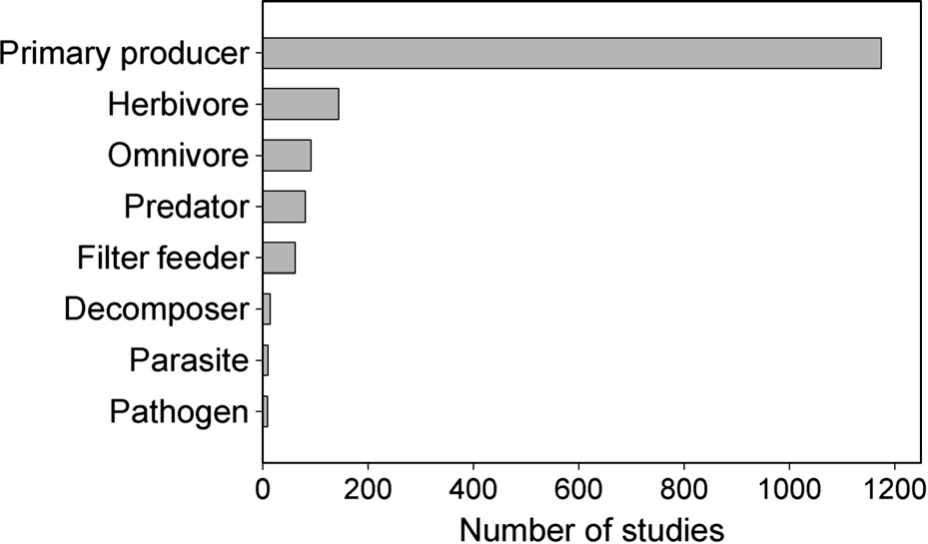
The trophic level of the introduced or invasive species that was principally investigated in each publication.

Almost three quarters of empirical studies (observational and experimental studies in the field, garden, greenhouse or controlled environments) were carried out on invaders that are primary producers (Fig. 7). Fewer than 10% of these studies were conducted on herbivores, while predators, omnivores, and filter feeders received much less attention. Only a small number of studies were published on pathogens, parasites, and decomposers.

**Figure 8:**
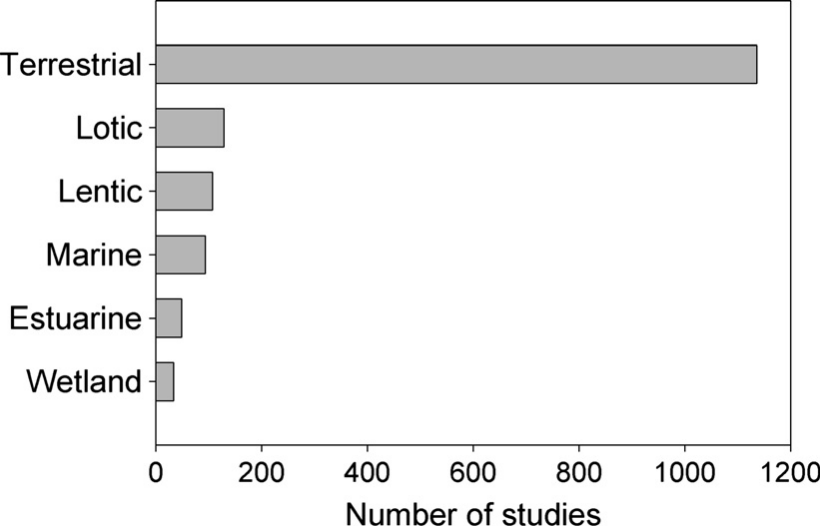
The type of ecosystem that was principally investigated in each publication.

Similarly, terrestrial systems were overwhelmingly represented among field studies (almost three quarters of field studies), with far fewer studies carried out in freshwater or marine systems, estuaries or wetlands (Fig. 8). This is confounded with trophic levels; of those studies conducted on terrestrial systems, 86% concerned plant invasions.

#### Invasion hypotheses

**Figure 9:**
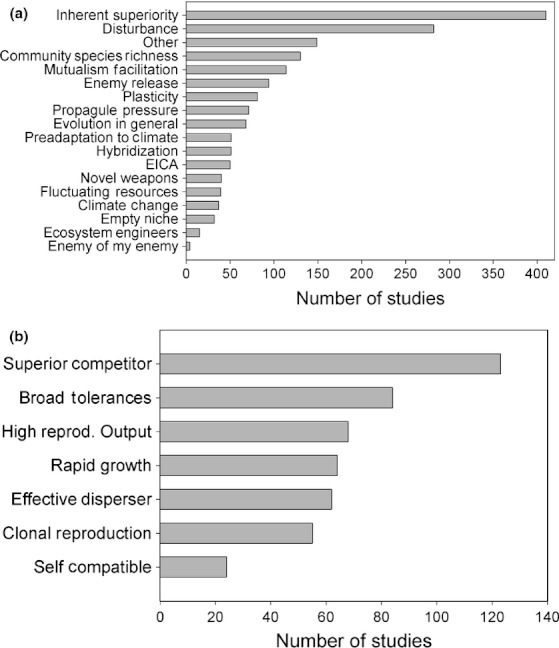
(a) The number of studies for each hypothesis that was evaluated. A description of the hypotheses is included in the Methods and described in more detail in Appendix 2. (b) In case the hypothesis evaluated was the “inherent superiority” of the competitive abilities of the introduced species, which characteristic of the invader (if specified) was responsible for its superiority.

We categorized the 1405 studies concerned broadly with evaluating hypotheses for invasions into 17 of the most common hypotheses explaining invasions, plus a category “other” for less common or less easily categorized hypotheses (Fig. 9a). Most of these papers were concerned with whether the data were consistent with the hypothesis, rather than attempting to test or disprove the hypothesis, or evaluating evidence for relative contributions of different causes of invasion, or for evidence in support of one hypothesis against another. The largest numbers of the studies concerned with invasion hypotheses focused explicitly or implicitly on the hypothesized inherent superiority of the invading species, followed by disturbance of the invaded habitat. While many of the studies examined hypotheses initially posed by Elton (1958), recent hypotheses (e.g., Evolution of Increased Competitive Ability (EICA), Novel Weapons, Ecosystem Engineers) were also well represented. Of these studies, 80% evaluated evidence for only one hypothesis, and 15% considered evidence for two hypotheses, while fewer than 4% of the studies considered or compared evidence in support of at least three hypotheses (Table 1). Approximately 20% of the reviews we included in our database were restricted to discussing a single-factor invasion hypothesis (e.g., biotic resistance, Levine et al. 2004; enemy release, Liu and Stiling 2006). Approximately 70% of the hypotheses tested were ecological or mostly ecological in focus, about 12% were largely evolutionary in nature, and about 8% could be categorized as having an evolutionary ecology focus.

**Table 1:** The number of hypotheses tested within each of the studies evaluating causes of biological invasions

Number of Hypothesis Tested	Number of Studies
1	1137
2	210
3	50
4	7
5	1

For those papers concerned with the hypothesis of inherent superiority of the invading species, the most common explanations for the superiority were, in order, competitive superiority, broad environmental tolerance, high reproductive output, rapid growth, dispersal ability, clonal reproduction, and self-compatibility (Fig. 9b).

#### Scopus results

The SCOPUS search initially returned 18,226 possibly relevant records. Approximately half of these were identified as duplications of the Web of Science papers found, leaving 9835 SCOPUS records, from which we created a subset of 652 records concerned with field experimental studies. Of these 652 records, 47 were found by examining titles and abstracts to be relevant to our review and not duplicated in the Web of Science search. We estimated how many papers would be added from a full evaluation of SCOPUS records as follows: As 7.2% (47/652 studies) of the papers we initially identified were found to be relevant, we could estimate that 7.2% of the remaining 9183 (9835 – 652) non-duplicate SCOPUS papers, or 661 additional papers, would be found to be relevant if we had the capacity to do a complete evaluation of these publications. As a second estimate, as 6.2% of the Web of Science papers initially identified in the process of carrying out the Field Synopsis were found to be relevant, if we instead use this number (0.062*9183 = 569), we obtain an estimate of a range of 569–661 papers that could potentially be added from a full evaluation of the uniquely identified papers in the SCOPUS database.

**Figure 10:**
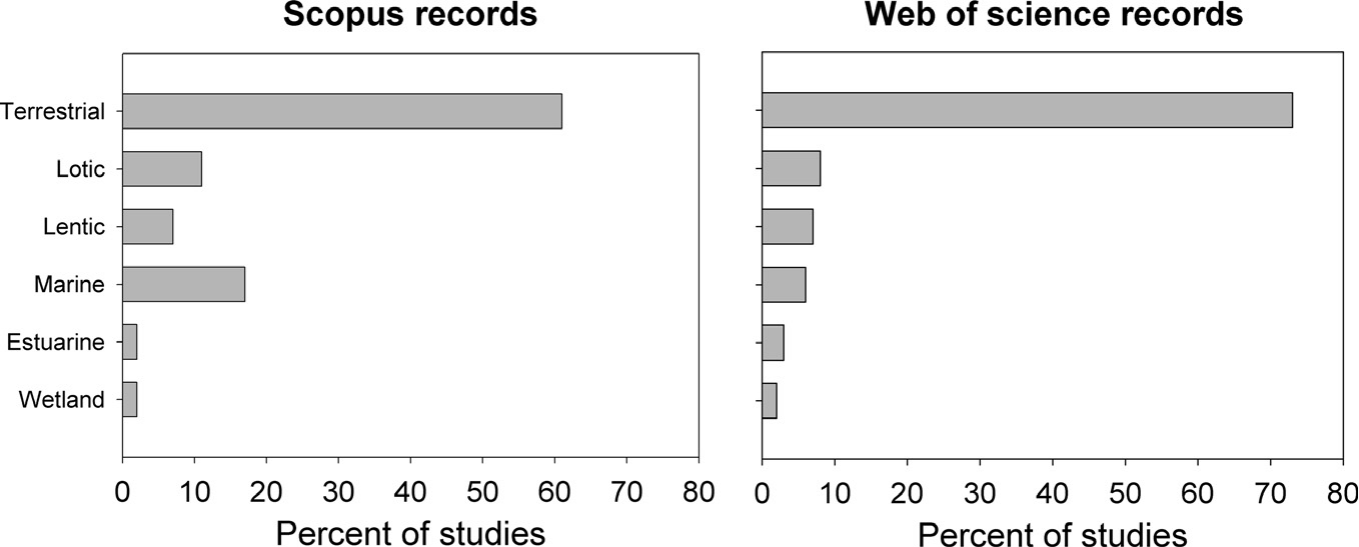
A comparison of the ecosystem that was principally investigated in publications that were found using the SCOPUS search service versus those found using the Web of Science service.

A comparison of these 47 papers unique to the SCOPUS search with the 312 experimental fieldwork studies that were identified in the Web of Science search indicates a greater representation of non-U.S.-based journals in the SCOPUS papers (albeit restricted to those in the English language by our search) that were not identified by Web of Science search. The journals included in SCOPUS appear mostly to be more narrowly restricted by field or geographically (e.g., the Japanese Journal of Limnology). There were also a somewhat larger proportion of marine publications in the SCOPUS results (e.g., Botanica Marina; Estuarine, Coastal and Shelf Science) in comparison with those in the Web of Science search (Fig. 10).

## Discussion

Biological invasions have received a great deal of scientific attention, with intense controversies, many hypotheses proposed, with important implications for fundamental understanding of the ecology and evolution of natural systems, for management, and for their ecological and economic impacts. This interest has produced a large literature, which, however, covers the field unevenly. The field synopsis and systematic review in this paper offer a broader and more comprehensive overview of the literature on biological invasions than has been available previously, and include approximately an order of magnitude more studies than previous reviews. The present paper is neither a conceptual framework, nor a meta-analysis. It does not quantify effect strength (e.g., the intensity of the impacts on native species, Vilà et al. 2006). We have attempted to scientifically describe and categorize what has been done and what has been studied about biological invasions, rather than proposing how to think about or study invasions, or about quantifying how invasions impact natives.

How does the approach taken here lead to new or different perspectives from other efforts to generalize about invasion biology? On the basis of the results of the field synopsis and systematic review, we can make several major generalizations about what is known about biological invasions from the published literature as identified in the ISI Web of Science (and partially in SCOPUS). Some of these are as follows: Scientific papers on biological invasions were very limited until the early 1990s, and expanded greatly in the late 1990s; we now have a systematically obtained quantitative assessment of the trajectory of this literature. A large proportion of the work that has been published is based on field observational studies; many fewer studies involve experimental work of all kinds (lab, field, etc.) or other types of investigations (e.g., theory or reviews). The pioneering work of Charles Elton remains a guiding presence in the field, strongly influencing the hypotheses tested, although new hypotheses are also addressed. Most studies are concerned with ecological rather than evolutionary questions. Terrestrial plant invasions are highly disproportionately represented in this literature. Information about biological invasions is available over a broad geographic range globally, but is focused on particular areas (e.g., North America, Western Europe, New Zealand), and there is a dearth of information (at least in English) about biological invasions in the tropics. Most studies evaluating hypotheses for invasions consider one or at most two hypotheses as possible explanations for invasions.

We are not able to address the pressing question of whether the foci of the studies effectively represent the occurrences or importance (in any sense of the term) of biological invasions, but we suspect that they do not. While there has been a great deal of interest in the importance of invasive predators and aquatic invertebrates, for example, this emphasis is not reflected in the proportions of published papers. Even if there is some bias in our ability to identify other literature (e.g., marine invertebrates), it is unlikely that this would change the overarching emphasis on terrestrial plant invasions in the literature. One can hypothesize many reasons for this: our search did not do a good enough job in locating papers from other areas (probably true, but this would almost certainly not change the overall picture of the emphasis on terrestrial plants in the literature); terrestrial plants may be easier and less expensive to study; reports on some important organisms or systems may be found disproportionately in the so-called gray literature (e.g., government reports) rather than in publications included in the Web of Science database; or there are a greater number of plant ecologists relative to ecologists who study other systems (although that begs the question of why this should be the case). The much greater amount that is known in terrestrial systems in contrast to marine ones also does not reflect the economic importance of marine and other less-studied systems.

While ecological explanations for invasion are far more likely to be investigated than evolutionary ones, we have no way of knowing if that reflects the prevalence of ecological rather than evolutionary causes of invasions, or merely of the prevalence of ecological rather than evolutionary scientists carrying out the studies. Designing studies that address ecological mechanisms or processes of invasion may be more tractable than evolutionary ones, given the respective time frames over which they occur. Applied ecological aspects of invasions are of more direct concern to the general public than evolutionary ones, and it seems probable that this could influence invasion biology research.

Biological invasions have been studied intensively in some locations and some systems and much less in others, but the degree to which the literature we identified accurately reflects the extent and importance of biological invasions in different systems and locations is completely unknown. Interestingly, our findings are only partially in accord with a recent study by Martin et al. (2012) on where ecologists carry out their research. Similar to their results, many of our studies were in temperate terrestrial environments. Likewise, they found underrepresentation of ecological research in Africa and South America, in agreement with our results on studies on invasions, but in contrast, they found overrepresentation (as defined by the proportion of geographic land mass) of studies in Central America, Greenland, and other areas in which we did not find a large number (or any) studies on biological invasions.

Undoubtedly, our results also reflect in part the restriction of our review to English language publications. For example, Rodriguez-Castañeda (2012) was able to greatly increase the number of tropical ecological studies in her meta-analysis by including papers published in Spanish and Portuguese. Not only are potentially vital instances or example of invasion going unexamined that could advance the science, but serious future ecological or economic harms may be unrecognized in understudied regions where the impact of human activities is increasing. This is a critical issue for both practical and theoretical reasons, and one which our results highlight in a way that may have been suspected but has not been previously quantified or highlighted.

Systematic reviews in the biomedical sciences (and to some extent in the social sciences) are intended to focus on narrow questions. By carrying out a very broadly defined review, our search identified so many relevant publications that we were not able to include several databases, or to fully categorize all of the papers we found. In addition, while medical databases are cross-referenced and so generally automatically exclude duplicate studies when searching more than one database, this was not the case for the ecological literature, and identifying and excluding duplicates were more challenging. The most serious limitation was that due to the time and effort constraints imposed by the very broad search, we were unable to use other means for identifying missing papers, such as combing the literature cited sections of papers.

Web of Science searches are idiosyncratic, and sometimes inexplicably fail to turn up portions of the literature, even when the search terms are included in the key words, etc. We know that we missed numerous papers in the search as it was defined, including some important and highly cited ones (e.g., Parker et al. 1999 was missed because the journal in which it was published is not indexed before 2004, when Web of Science adopted it) and even some of our own publications (e.g., Gurevitch and Padilla 2004). Undoubtedly, these flaws resulted in not only omissions, but also biases in the larger picture of the literature; the extent to which these biases exist and color the results are unknown, and it will take further extensions of this effort to better understand and more importantly, correct them.

Although our study suffers from limitations, we believe that the conclusions of our research synthesis and the database produced are excellent starting points for future research. Other studies can build on our findings and make advances by correcting the errors we made in carrying this review out. These limitations also do not diminish the great value of the systematic review approach; all other methods of searching the literature suffer from the same problem, but also from many other limitations such as unintended bias and lack of transparency.

Systematic review methodology offers many advantages over previous research synthesis methods for ecology, evolution and conservation biology. The results can be better and more accurately evaluated, investigated, and updated. These are some of the reasons it has become standard in medicine and other disciplines. One of the ways in which bias is reduced in other fields is that systematic reviews may be registered before being conducted, with strict protocols detailed in advance and followed during the review process (e.g., see Higgins and Green 2009). There have been efforts to adopt this practice in the field of conservation biology (e.g., Stewart et al. 2009; Stewart 2010), but it has not yet been incorporated more broadly in ecology and evolutionary biology, where people often want to change the direction of either primary research or reviews depending on where the findings of the study lead. While a really large scope field synopsis or very broad systematic review such as this one may not be attempted very often in these fields, our review and database may serve as a foundation for future reviews. We believe that systematic review methodology should be much more widely adopted in these fields because of its obvious scientific basis and many advantages.

## Appendix 1

Systematic review standards and practices in other disciplines and limitations of the search.

The distinction between systematic reviews, field synopses, and scoping reviews tends to be somewhat blurred (e.g., Mocellin et al. 2012). What we have called a systematic review might be considered a “scoping review” or “systematic map” in some disciplines, rather than a systematic review, because it is not focused on a single, narrowly specifically defined question.

Ideally at least two readers should evaluate all studies in a systematic review. We did not do this rigorously because we were not fully aware of all of the protocols in other disciplines when we began and carried out our review, and for practical reasons (limited person-hours were available for this largely volunteer study). The Cochrane and Campbell Collaborations now require consultation with information specialists/librarians in coming up with search strategies (Julia Littell, pers. comm.); we recommend this for future systematic reviewers in ecology and evolution, but were unaware of this practice at the time of carrying out this review.

Although we were not able to follow all of the established methodology that has been developed in medicine and the social sciences for systematic reviews (e.g., we did not formally assess the reliability of coding, we only used two databases, we did not work with research librarians/information specialists) we strongly encourage researchers carrying out future systematic reviews in ecology and evolution to develop and follow such guidelines as appropriate for the fields of ecology and evolution; e.g., based on the PRISMA standards, http://www.prisma-statement.org/ or the MOOSE standards (Stroup et al. 2000); and see the EQUATOR network guideline summaries, http://www.equator-network.org/home/ and the NIH summary, http://www.plosmedicine.org/article/info%3Adoi%2F10.1371%2Fjournal.pmed.1000217).

One of the greatest challenges in any review, including a systematic review, is missing papers. We acknowledge that we have missed papers. Even in a systematic review, while the methodology is transparent, there are various things that make it not fully repeatable, although it is far more repeatable than for a traditional review. In such a large review, omissions are even more likely. Some reasons include that the search engine changes its algorithms, includes additional journals at later dates, and has a lag between publication of papers and inclusion in the database (sometimes a fairly long lag). The sequence by which papers are identified and excluded alters the outcome. Other failures to identify papers are more difficult to understand. In addition, there is a subjective element to the decision to include or exclude papers based on their content and topic.

### Literature cited for Appendix 1

Mocellin et al. 2012. Telomerase Reverse Transcriptase Locus Polymorphisms and Cancer Risk: A Field Synopsis and Meta-Analysis. J. Natl. Cancer Inst. 104:840–854.

Stroup et al. 2000. Meta-analysis of observational studies in epidemiology: a proposal for reporting. Meta-analysis Of Observational Studies in Epidemiology (MOOSE) group. JAMA 283:2008–2012.

## Appendix 2

Information collected from records found in the search, and categories used in classifying studies. The first item was used in categorizing studies in the field synopsis (focus and type of research) and the remaining items were used in the systematic review.

**Table:**

Information collected from studies	Classifications within each category of information
Focus of the work	Hypothesis about invasions examined (implicitly or explicitly) Impacts of invasions Testing fundamental ecological ideas with invasive systems
Type of research	Field –experimental Field – observational Theoretical/modeling Statistical/meta-analysis Greenhouse Garden Lab Review
Invader species name	
Trophic level of invader	Primary producer Herbivore Predator Omnivore Decomposer Filter feeder Pathogen Parasite
Location of invasion under study	Country, state, local area name (i.e. parkland, lake or river) If given: Latitude and Longitude
Ecosystem	Terrestrial Marine Lentic Lotic Wetland Estuarine
Biome	Grassland Deciduous forest Coniferous forest Tropical forest Subtropical forest Urban/old field Savanna Chapparal/shrublands Wetland/riparian Mountain/alpine Tundra Intertidal/near shore Pelagic/open ocean Coral reef Benthic
Hypothesis considered by study	Climate Change Community Species Richness Disturbance Ecosystem Engineers Empty Niche Enemy Of My Enemy Enemy Release Evolution of Increased Competitive Ability (EICA) Evolution in General Fluctuating Resources Hybridization Inherent Superiority (Ideal Weed) Mutualism, Facilitation, or Invasional Meltdown Novel Weapons/Allelopathy Plasticity Preadaptation to Climate Propagule Pressure Other
If the hypothesis being considered is the “inherent superiority” of the invader, mechanism postulated for superiority	Broad Tolerances Clonal reproduction Effective disperser High Reproductive output Rapid Growth Self compatible Superior competitor

## Appendix 3

Explanations of hypotheses examined in Appendix 2 and Figure 9. Note that authors may not have explicitly identified a hypothesis, and that we generalized specific ideas being tested so that we could categorize the literature. For example, a study hypothesizing that the success of an invading species was due to higher photosynthetic rates or superior competitive abilities would be categorized as evaluating its “inherent superiority” (Hypothesis 12)

**Table:**

1. Climate change
Changing climate patterns contribute to invasion.
2. Community species richness
The process of invasion is affected by community species richness.
3. Disturbance
Alteration of the habitat due to natural phenomena (fire, mudslides, flooding etc.) or due to human disturbances contributes to invasion.
4. Ecosystem engineers
The invasive species alters the environment in a way alters ecosystem function, niche structure or the competitive landscape.
5. Empty niche
The invasive species uses resources that are unexploited in the invaded range.
6. Enemy of my enemy
A third species interacts with a negative effect on native species in the introduced range, contributing to the success of an invasive species.
7. Enemy release
The invasive range of a species may not include the natural enemies or similar organisms that limited its populations in the native habitat.
8. Evolution in general
The invasive species evolves to become different from the native ancestor (due to various responses to selection or other evolutionary changes, but distinct from EICA and other specific evolutionary hypotheses listed here).
9. Evolution of increased competitive ability (EICA)
Due to the relaxation of predation or herbivory, the invading species may evolve traits that permit it to become a better competitor in the invasive range than in the native range.
10. Fluctuating resources
Ability to exploit repeated changes in resource levels permits an introduced species to become an invader.
11. Hybridization
The invasive species may be the product of intraspecific hybridization between populations from different parts of the native range, or interspecific hybridization with other species native to the invaded or any other area.
12. Inherent superiority
The invasive species possesses traits that make it superior (due to particular traits may be specified).
13. Mutualism, facilitation or invasional meltdown
Another organism in the novel environment facilitates the success of the invasion.
14. Novel weapons
The invasive species has characteristics that negatively affect the species it interacts with in the introduced range in the specific ways identified explicitly by this hypothesis.
15. Plasticity
An invasive species has a highly plastic phenotype that is capable of enhanced response to environmental conditions (often resource levels) found in an introduced range, that contributes to its establishment or competitive success.
16. Preadaptation to climate
The existing environmental tolerances of an introduced species allow it to become invasive in the matching environmental conditions in a new range.
17. Propagule pressure
Invasion is the result of a large number of propagules being introduced to the invaded environment.
18. Other
Any other hypothesis on invasions not defined above.

## Appendix 4

eadsheet with the database including all the publication records that were collected. This includes all results after an initial screening of title and abstract, but before evaluation using the full text, and includes the SCOPUS records

## Appendix 5

Spreadsheet with the database of the publication records that were used in the field synopsis

## Appendix 6

Spreadsheet with the database of the publication records that were used in the systematic review
